# Endoscopic Management of Unusual Bezoar in a Prader-Willi Syndrome Patient

**DOI:** 10.7759/cureus.29900

**Published:** 2022-10-04

**Authors:** Ayah Awawda, Hazem Al Ashhab, Isam Shammas, Mohammad Al Mohtasib, Yousef Abu Asbeh

**Affiliations:** 1 Faculty of Medicine, Al-Quds University, Jerusalem, PSE; 2 Gastroenterology, Al-Quds University Medical School, Jerusalem, PSE; 3 Surgical Intensive Care Unit, Al Ahli Hospital, Jerusalem, PSE; 4 General Surgery, Al Ahli Hospital, Jerusalem, PSE; 5 Thoracic Surgery Unit, Al Ahli Hospital, Hebron, PSE

**Keywords:** bezoar, prader-willi syndrome, gastric bezoar, endoscopic approach, melena, threads bezoar

## Abstract

Bezoar occurs due to the ingestion of inedible material. The most common bezoar is a phytobezoar, which results from the ingestion of indigestible food particles found in vegetables and fruits. Other types include trichobezoar, which involves hair, lactobezoar, which involves milk products, pharmacobezoar, which involves medication, and in unusual cases, bezoar may involve different materials such as metals, plastics, and paper. We are presenting a case of a 19-year-old patient, a known case of Prader-Willi syndrome, who presented with difficulty breathing and tachypnea after aspiration of grape particles, and then he started to complain of melena and vomiting of dark content. He was admitted for urgent bronchoscopy and endoscopy, which showed a bezoar composed of grapes and threads. He was managed endoscopically by removing most of the threads and grape particles and releasing the tangled threads to facilitate its migration distally. Follow-up endoscopy showed complete resolution of the previously noticed content. We reported this case to discuss the endoscopic management of unusual bezoar involving threads.

## Introduction

Gastrointestinal (GI) bezoars are a collection of inedible material found in the GI tract. Bezoar has multiple types, most commonly phytobezoar, which occurs due to the ingestion of indigestible food particles found in vegetables and fruits. Trichobezoar occurs due to the ingestion of hair. Lactobezoar involves milk products. Pharmacobezoar involves medication, and in unusual cases, bezoar may involve different materials such as metals, plastics, and paper. Bezoar could occur due to multiple predisposing factors, for example, gastric operation, mental retardation, psychiatric disorder, coeliac disease, pica, and neurodevelopmental disease. Patients may present differently depending on the type of bezoar, size, and site. Treatment of bezoar could involve multiple approaches such as dissolving, endoscopic approach, and surgical intervention [1].

## Case presentation

A 19-year-old male patient, a known case of Prader-Willi syndrome, associated with obesity and hypotonia, complained of difficulty breathing for a one-week duration. Before one week, an aspiration of grapes occurred while the patient was eating, after that he started to complain of difficulty breathing and tachypnea, associated with fever. He was managed with O2 and antibiotics. After that, he started to complain of melena and vomiting dark content. He was then referred to our hospital for bronchoscopy and endoscopy. Clinical examination revealed stridor with inspiratory wheezing, crepitation bilaterally, mainly at the left side, tachypnea, difficulty breathing, and decreased air entry on the left side.

**Figure 1:**
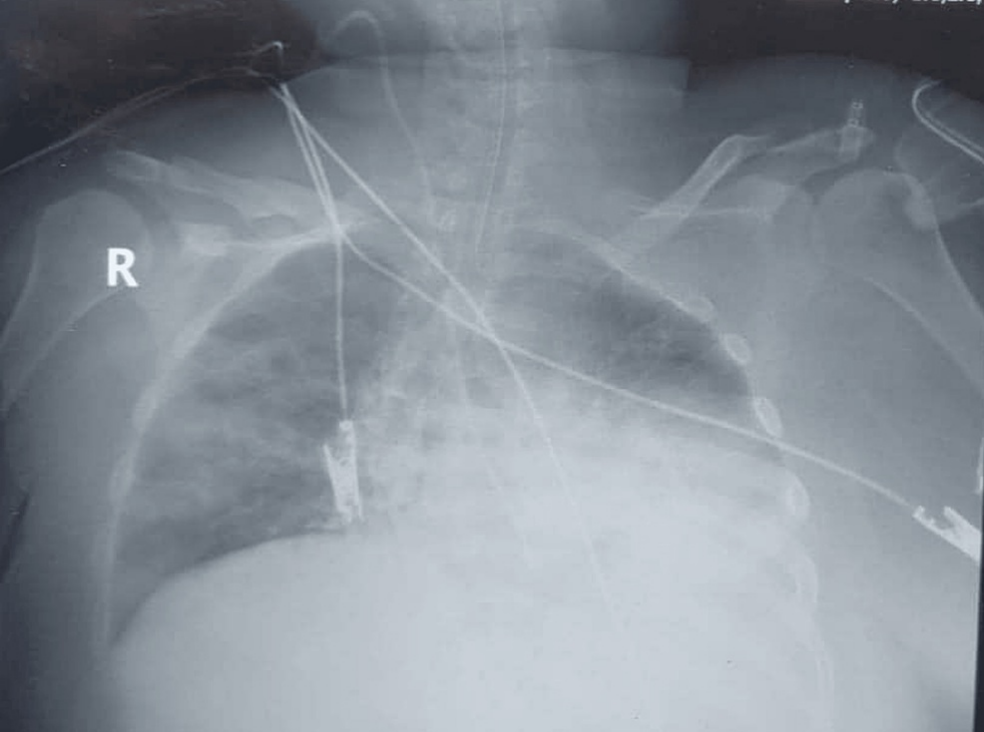
Patient's chest X-ray on admission showing left lower lobe infiltration

Laboratory evaluation showed a decreased hemoglobin level of 7.62 g/dl, decreased red blood cell count of 2.7 × 10^6, decreased hematocrit level of 27.6 pg/mL, and elevated white blood cell count of 67.5 × 10^3. Clinical chemistry showed a decreased calcium level of 7.9 mg/dl, normal electrolytes, elevated C-reactive protein of 82.4 mg/l, elevated blood urea nitrogen (BUN) level of 41 mg/dl, and normal creatinine level (Table 1). A chest X-ray showed left lung infiltration (Figure 1).

**Table 1:** Patient's labs upon admission

Test	Result	Reference range	Unit
Hemoglobin (Hbg)	7.62	Male: 12.6-17.5; female: 12.0-16	g/dl
Red blood cells (RBC)	2.6 * 10^6	(4.6-6.2) * 10^6	/mm^3^
White blood cells (WBC)	67.5 * 10^3	(5.0-10.0) * 10^3	/mm^3^
Hematocrit (Hct)	27.6	40-52	%
Calcium (Ca^+2^)	7.9	8.5-10.4	mg/dl
C-reactive protein (CRP)	82.4	Up to 6	mg/l
Blood urea nitrogen (BUN)	41	4.7-23.4	mg/dl
Creatinine (Cr)	0.79	Children: 0.2-1.0; adult – males: 0.4-1.2; over 60 years old: 0.6-1.5	mg/dl

The patient was admitted to the ward, after that his oxygen saturation reached 75% on room air, and we decided to transfer him to the surgical intensive care unit, where he was intubated, mechanically ventilated, and an arterial line and Foley catheter were applied, and he was started on IV antibiotic ceftriaxone.

**Figure 2:**
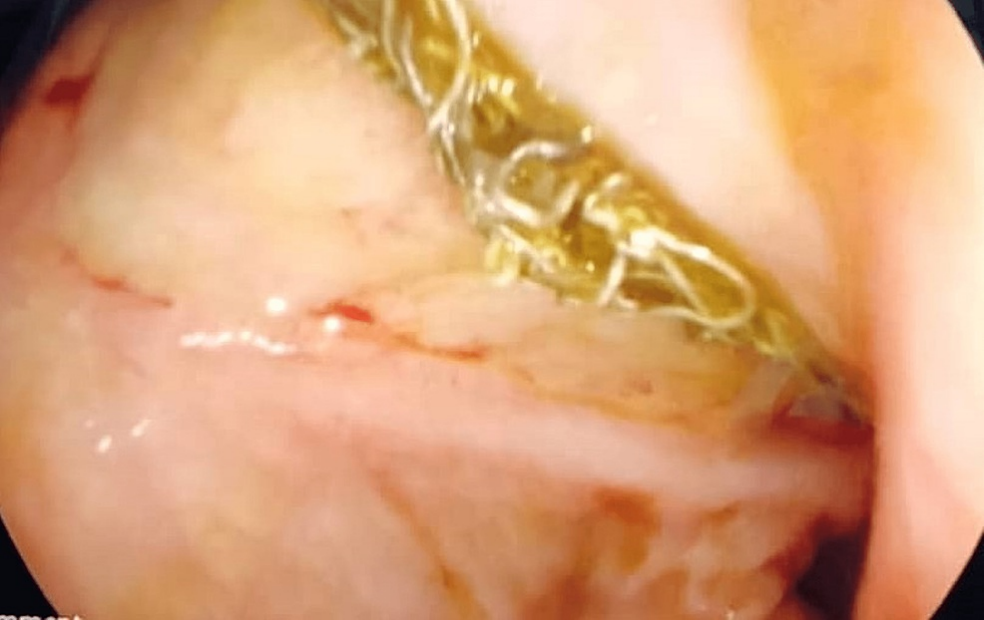
Endoscopy showing thick threads in the esophagus

**Figure 3:**
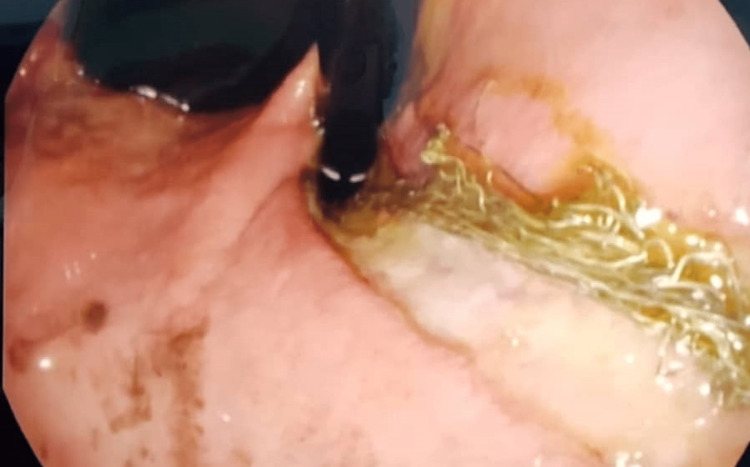
Retrograde endoscopy showing stomach pylorus and fundus with thick threads and ulceration

Urgent bronchoscopy and upper GI endoscopy were performed. Bronchoscopy showed an edematous vocal cord. Thick secretions were found in the right and left lower lobe bronchus, and no evidence of a foreign body was found. On the other hand, upper gastrointestinal endoscopy showed a bezoar that was composed of grape particles and a thick bundle of threads. The threads were arranged in a continuous bundle extending from the upper esophageal sphincter to the fourth part of the duodenum in a contentious and connected manner (Figures 2, 3).

A major part of the threads was removed during endoscopy, but there was difficulty removing some threads due to distal resistance and continuity distally to the small bowl. So we elected to remove most of the threads using the endoscopic foreign body removal, or the alligator forceps. After we removed 70% of the threads, we elected to cut the thread bundle using endoscopic scissors, allowing the threads to be released distally, and removing the proximal part. Extensive areas of pressure necrosis were noticed from the upper esophagus extending down to the third part of the duodenum, and no evidence of full-thickness perforation was noticed in the esophagus, stomach, or duodenum.

The patient was referred to surgical ICU, where the patient was kept nil per os (NPO) and a septic evaluation was done, which showed *Escherichia coli* in the nasal swab. His antibiotic was upgraded to IV piperacillin/tazobactam 4.5 mg × 4 and IV fluconazole with 400 mg loading dose and then 200 mg daily for the fear of the presence of micro-perforation, with respiratory physiotherapy.

**Figure 4:**
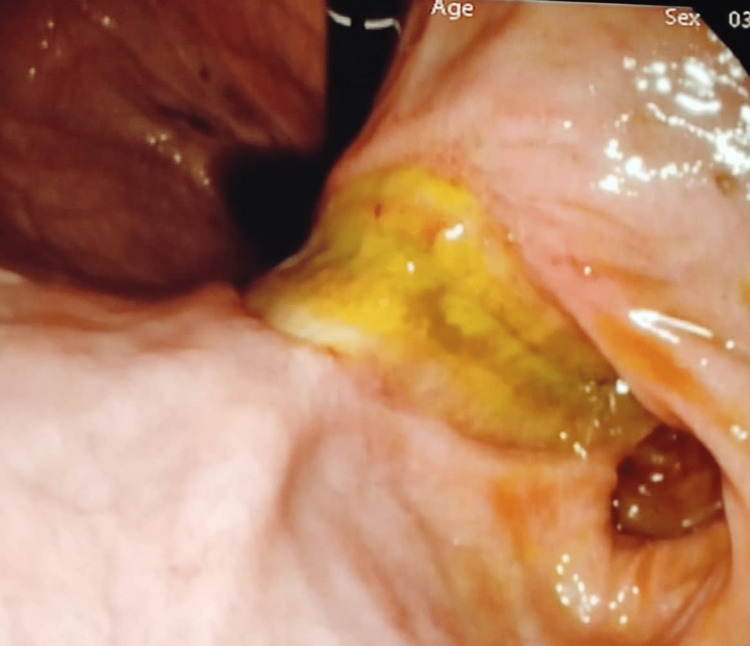
Follow-up endoscopy showing stomach pylorus and fundus without ulceration and threads

The patient was stable following the procedure. A follow-up bronchoscopy and upper GI endoscopy were performed 12 hours later. Bronchoscopy was non-remarkable. Endoscopy showed that the previously noticed threads were no longer noticed, ulceration was noticed on the esophagus and stomach wall, and no evidence of perforation was noted (Figure 4).

**Figure 5:**
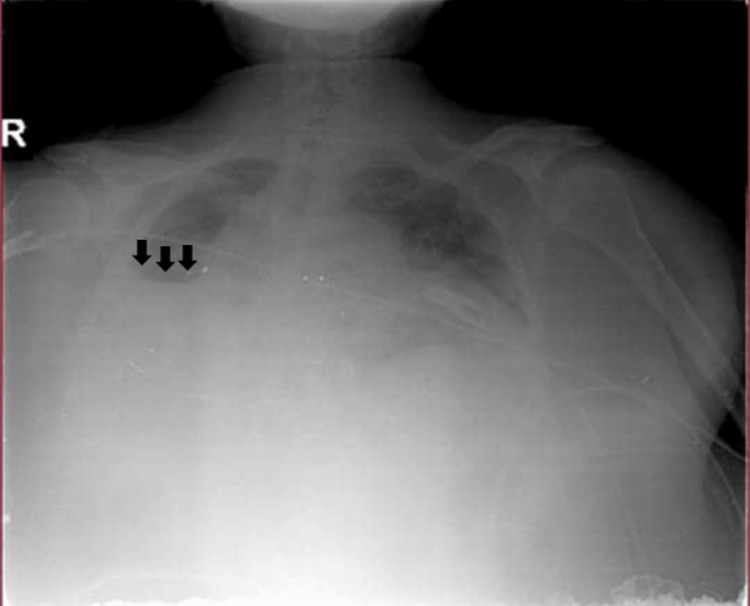
Chest X-ray after unfortunate aspiration. Arrows represent infiltration on the right side

**Figure 6:**
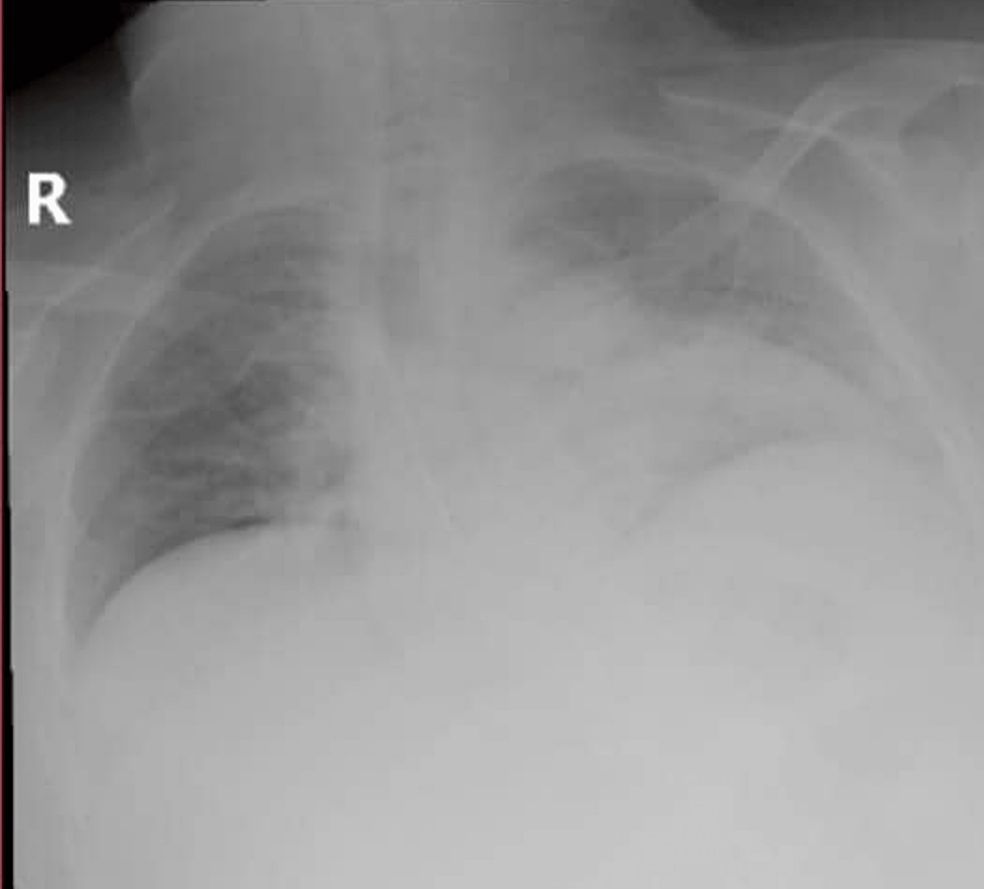
Patient's chest X-ray on discharge

The patient continued to be evaluated in the surgical ICU and was extubated on the second day. He was then transferred to the ward, where an unfortunate aspiration occurred. A chest X-ray was done showing infiltration on the right side, and it was confirmed by ultrasound (Figure 5), which lead him to be readmitted to the surgical ICU, where he was managed by piperacillin/tazobactam (4.5 mg × 4) and vancomycin (1 gram × 2), with respiratory physiotherapy. He continued to excrete threads with the stool, which is associated with melena, and stopped seven days after admission. The patient was discharged home after a notable improvement in his labs and his clinical condition (Figure 6). Psychiatric support was sought initially, but there was limited compliance due to a lack of resources.

## Discussion

Bezoar occurs when inedible material got ingested. There are many predisposing factors of bezoars, including dietary behavior and previous gastric surgery. Predisposing factors differ from adults to children. In adults, a bezoar is commonly associated with gastric operations; in children, it is related to mental retardation, psychiatric disorder, coeliac disease, and pica, but it could also be associated with neurodevelopmental diseases like Prader-Willi syndrome, which is a genetic disease occurring due to defect in gene expression on the paternally inherited chromosome 15, which affects multiple body systems and induces psychiatric disorder. It is also associated with hyperphagia and obesity. Our patient had Prader-Willi syndrome and suffered from obesity and hyperphagia, which led him to frequently ingest threads [2].

The clinical presentation of bezoar depends on different factors, and it varies based on the type. Most bezoars have a common presentation; they usually present with nausea, vomiting, and abdominal pain, but some of them could have a special presentation. Lactobezoar may present with feeding difficulties, distension, irritability, or palpable abdominal mass. Pharmacobezoar may present gastric outlet obstruction, but it varies based on the pharmacological composition. Trichobezoar may present with gastric outlet obstruction, ulceration, GI bleeding, and perforation. Phytobezoar can present as gastric outlet obstruction [1].

The size and sites could affect the presentation. Mostly bezoar is located in the stomach, jejunum, and ileum. Some patients can be completely asymptomatic [3]. Our patient's initial presentation was an aspiration, but then he developed melena, which was a result of a bezoar composed of threads that were adherent to the wall of the esophagus and duodenum, causing ulceration and bleeding.

Bezoar is diagnosed by history, clinical examination, and radiological investigation, which play a major role in diagnosis. Bezoar can appear as an opaque mass, but difficulties could rise in some cases where bezoar was a radiolucent material. The initial chest X-ray in our case showed lung infiltration in the left lower lobe, and it did not show any foreign material.

Endoscopy is considered the best diagnostic approach that can be used in bezoar cases [2]. In our patient, endoscopy showed a bezoar that was composed of grape particles and a thick bundle of threads that extended from the esophagus to the duodenum and they were adherent to the wall causing linear ulceration and significant dilation of the distal part of the duodenum and area of penetration within the wall.

In bezoar treatment, our goal is to remove the bezoar and prevent a recurrence. Managing varies based on the type of bezoar. Managing the phytobezoar is divided into three categories: dissolution, fragmentation, and retrieval. There are multiple techniques, and these techniques can be used individually or combined. Endoscopic therapy mainly focuses on mechanical disruption. Surgical removal is restricted for patients who failed previous treatment options, or developed complications such as bleeding or perforation. Trichobezoar is enzymatic resistant; therefore, the only option is surgical treatment, although endoscopic removal has been successful in some cases. Lactobezoar is typically managed conservatively. Pharmacobezoar treatment depends on the pharmacological structure and the patient’s clinical situation. Treatment options can vary from dissolution therapy and bowel irrigation to endoscopic or surgical removal in severe cases [1].

Threads are resistant to enzymatic dissolution; therefore, the only option in treatment is surgical removal, but some enzymatic-resistant bezoars like trichobezoar are reported to be managed via endoscopy. A case report from 2011 presented a nine-year-old girl with gastric trichobezoar successfully treated by endoscope using a polyp retrieval. Endoscopic removal is cost-effective and less invasive than surgical removal [4]. So we considered endoscopy as an option for treatment in our patient. We removed most of the threads using the endoscopic foreign body removal and the alligator forceps. After we removed 70% of the threads, we elected to cut the thread bundle, allowing the threads to be released distally, and removing the proximal part.

## Conclusions

Bezoar has different types. Some of them need surgical treatment like trichobezoar and other unusual materials. We reported a patient with Prader-Willi syndrome as a case of threads bezoar who presented with melena and was successfully managed by endoscopic removal. Endoscopic removal is less invasive and cost-effective. It has fewer complications and is more cosmetically appealing than surgical removal. Thus, endoscopy should be considered as a way of unusual bezoar treatment. Also, psychiatric evaluation and endoscopic follow-up should be advised for patients who have predisposing factors for bezoar.
